# Isolation and comparative genomics of *Mycobacterium tuberculosis* isolates from cattle and their attendants in South India

**DOI:** 10.1038/s41598-019-54268-x

**Published:** 2019-11-29

**Authors:** Kannan Palaniyandi, Narender Kumar, Maroudam Veerasamy, Ahmed Kabir Refaya, Chandrakumar Dolla, Subramanyam Balaji, Dhanaraj Baskaran, Kannan Thiruvengadam, Ananthi Rajendran, Sujatha Narayanan, Dhinakar Raj, Soumya Swaminathan, Sharon J. Peacock

**Affiliations:** 1 ICMR-National Institute for Research in Tuberculosis, Chennai, India; 2 Department of Medicine, University of Cambridge, Hills Rd, Cambridge, CB2 0QQ United Kingdom; 3 Translational Research Platform for Veterinary Biologicals (TRPVB), Tamil Nadu Veterinary and Animal Sciences University, Chennai, India; 4 World Health Organization, Geneva, Switzerland

**Keywords:** Clinical microbiology, Pathogens

## Abstract

The major human pathogen *Mycobacterium tuberculosis* is rarely reported to cause disease in other animals. Cases in livestock are thought to occur through contact with infected handlers, but previous studies evaluating putative livestock-human transmission used typing techniques with limited resolution. Here, we undertook cross-sectional surveillance for tuberculosis in 271 livestock handlers and 167 cattle on three farms in Chennai, India and defined the relatedness of cultured isolates using whole genome sequencing. Humans and livestock were screened for active mycobacterial infection, and opportunistic post-mortem examination was performed on comparative intradermal test-positive cattle that died. Four cattle and 6 handlers on two farms were culture-positive for *M*. *tuberculosis*; *M*. *bovis* was not isolated. All 10 isolates (one from each case) belonged to Lineage 1. Pairwise genome comparisons of single nucleotide polymorphism (SNP) differences ranged from 1 to 600 SNPs, but 3 isolate pairs were less than 5 SNPs different. Two pairs were from handlers and the third pair were from two cattle on the same farm. The minimum pairwise SNP difference between a cattle and human isolate was >250 SNPs. Our study confirms the presence of *M*. *tuberculosis* infection in cattle in India, sequencing of which characterised relatedness between human and cattle-derived isolates.

## Introduction

*Mycobacterium tuberculosis* and *Mycobacterium bovis* are the predominant cause of tuberculosis in humans and cattle, respectively^1^. *M*. *bovis* can also infect humans following direct contact with cattle (via aerosols) or from the consumption of raw milk^2^, control of which includes culling of infected cattle and pasteurisation of milk^3^. *M*. *bovis* has a wide host range, and control of bovine tuberculosis includes control of vector animals that may form part of a transmission chain between cattle^3^. By contrast, *M*. *tuberculosis* infection is primarily restricted to humans with limited transmission to other animals, with the majority of reported cases occurring in cattle^4–6^.

Non-human *M*. *tuberculosis* infection is considered to represent accidental infection of animals in close proximity to infected humans, often in regions where human tuberculosis is common^6,7^, with no evidence of animal-to-animal transmission. This is largely based on epidemiological studies, with limited use of bacterial typing to determine the genetic relatedness between isolates from livestock and their handlers. In a study using IS*6110*-based restriction fragment length polymorphism (RFLP) typing, *M*. *tuberculosis* isolated from cattle and a handler had an identical pattern, suggesting transmission between them^7^. *M*. *tuberculosis* has also been isolated from cattle in ﻿Wuhan City in China, which when analyzed alongwith with TB patient isolates from the local hospital (including cattle handlers) identified them as the locally dominant Beijing type using spoligotyping^8^. Further investigation with MIRU-VNTR revealed that the patterns of the cattle isolates were identical to some of the TB-patient isolates, suggesting a possible link between the two^8^.

Although spoligotyping and VNTR have been used extensively to determine the relatedness of mycobacterial species, including isolates associated with disease involving livestock^6–9^, these methods are being increasingly replaced by whole genome sequencing. This provides higher resolution and when combined with epidemiological investigation can accurately determine transmission networks and outbreaks^10,11^. Whole genome studies could also be used to define sources of *M*. *tuberculosis* acquisition by livestock. Here, we describe an observational study of *M*. *tuberculosis* infection in cattle and their handlers at 3 farms in Chennai, India, in which we integrated the findings of an epidemiological investigation with bacterial culture, identification and whole genome sequencing.

## Results

### Participant recruitment, screening and culture

We identified 271 livestock handlers and 167 cattle on three farms in Chennai, India who were screened for tuberculosis. Fifty handlers (18.5%) had one or more symptoms that were consistent with tuberculosis (cough for >2 weeks, fever, night sweats and/or weight loss). These individuals had sputum samples taken for culture, 6 of whom were culture-positive for *M*. *tuberculosis*. Twenty-one cattle (12.6%) were positive for the comparative intradermal test (CIT) (Table 1), four of whom died during the study period and underwent post-mortem examination. All four had extensive visible tuberculous lesions in the lungs, spleen, liver and lymph nodes. Lesions typically appeared yellowish, caseous and necrotic with grey fibrous tissue. Pre-scapular and mesenteric lymph nodes contained calcified tubercles, lung tissue had palpable tubercles ~0.5 to 1.0 cm in diameter, and two animals had tuberculous mastitis. Lung tissue samples from all 4 animals were smear positive for acid-fast bacilli on ZN staining. Cultured isolates were identified as *M*. *tuberculosis* based on spoligotyping; *M*. *bovis* was not isolated. The human and livestock cases of tuberculosis occurred on two of the three farms (termed farm 1 and farm 2). Phenotypic susceptibility testing of the 10 isolates showed that 9 were susceptible to rifampicin, isoniazid, streptomycin and ethambutol, and one isolate (from cattle) was resistant to isoniazid and rifampicin (Table 2). Spoligotyping classified all isolates as East-African Indian (EAI) spoligotype Lineage 1 (Table 2), which is prevalent in South India. Efforts to isolate mycobacteria from nasal swabs and milk from CIT-positive cattle failed due to heavy contamination.

**Table 1:** TB screening of cattle and their handlers on three farms in Chennai.

Farm	CIT* testing of cattle		Screening of livestock handlers		
	Number screened	Number positive	Number screened	**Features consistent with TB	Culture-confirmed TB
Farm 1	21	2 (9.5%)	68	13 (19.1%)	2 (2.9%)
Farm 2	65	15 (15%)	186	35 (18.8%)	4 (2.2%)
Farm 3	81	4 (4.9%)	17	2 (11.8%)	0
TOTAL	167	21 (12.6%)	271	50 (18.5%)	6 (2.2%)

*Comparative Intradermal Test.

**Presence of symptoms consistent with TB (Cough, fever, night sweats & weight loss).

**Table 2:** Spoligotyping and drug susceptibility of *M*. *tuberculosis* isolated in this study.

Isolate ID	Source	Sample type	Farm	Spoligotype	Phenotypic susceptibility profile*
AH 03	Attendant	Sputum	Farm 1	EAI3	SSSS
AH 29	Attendant	Sputum	Farm 1	EAI3	SSSS
AH 69	Attendant	Sputum	Farm 2	EAI3	SSSS
AH 85	Attendant	Sputum	Farm 2	EAI3	SSSS
AH 90	Attendant	Sputum	Farm 2	EAI3	SSSS
AH 91	Attendant	Sputum	Farm 2	EAI2	SSSS
KH 126	Cattle	Lung	Farm 2	EAI5	SSSS
KH 127	Cattle	Lung	Farm 2	EAI5	SSSS
KH 128	Cattle	Lung	Farm 2	EAI5	SSSS
KH 143	Cattle	Lung	Farm 1	EAI5	SRRS

*Drug susceptibility profiles are in the following order: streptomycin, isoniazid, rifampicin ethambutol. S, susceptible; R, resistant.

### Genomic analysis of resistance and relatedness

**Figure 1:**
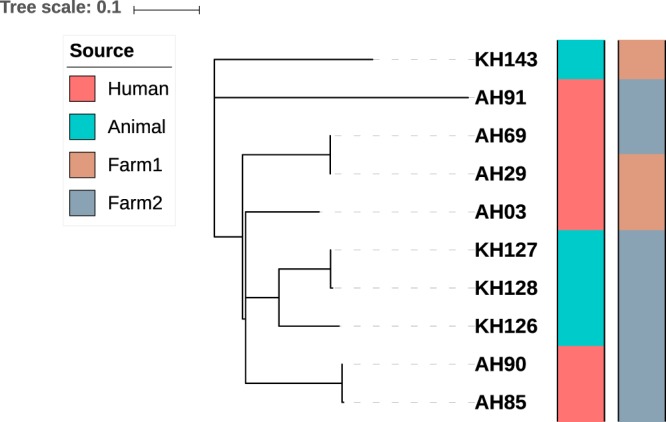
Phylogenetic tree of the 10 study *M*. *tuberculosis* isolates. Constructed using whole genome SNPs identified after alignment to the H37Rv reference genome and generated using RAxML with 1000 bootstraps. Tree scale of 0.1 represents ~123 SNPs.

**Figure 2:**
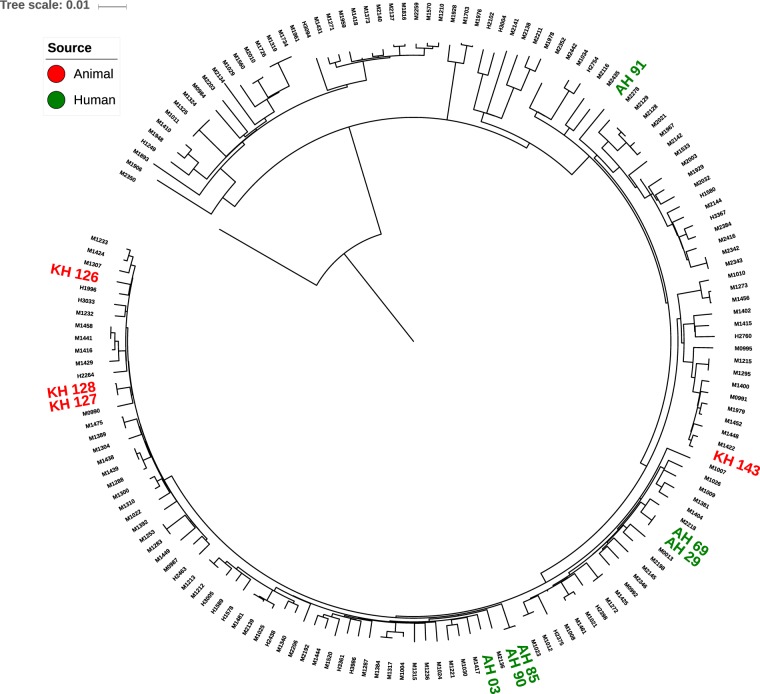
Contextual phylogenetic tree combining the 10 study *M*. *tuberculosis* isolates with a further 151 lineage 1 *M*. *tuberculosis* isolates from India. Constructed using whole genome SNPs identified after alignment to the H37Rv reference genome and generated using RAxML with 1000 bootstraps. The strain M2350 belonging to lineage4 was used as an outgroup. Study isolates are shown as red or green based on source. Tree scale of 0.01 represents ~168 SNPs.

Genomes of the 10 *M*. *tuberculosis* isolates were screened for the presence of mutations associated with resistance to isoniazid, rifampicin, streptomycin and ethambutol. This revealed mutations in 1 isolate (S/450/W mutation in *rpoB* and S/315/T mutation in *katG* conferring resistance to rifampicin and isoniazid, respectively), consistent with the phenotypic results. All 10 isolates were assigned to lineage 1 based on the presence of regions of difference (deleted RD239) and lineage 1 specific SNPs^12^, which is consistent with our spoligotyping results. A whole genome SNP based phylogenetic tree was constructed to evaluate the genetic relatedness among the study isolates, which demonstrated several clusters (Fig. 1). Pairwise comparison of the 10 genomes identified a SNP difference that ranged from 1 to 600 SNPs, but 3 isolate pairs were less than 5 SNPs different (Table 1). Pair 1 (isolates AH85 and AH90) from two handlers at farm 1 differed by 3 SNPs; pair 2 (KH127 and KH128) from two cattle at farm 2 differed by 4 SNPs; pair 3 (AH29 and AH69) from two handlers at different farms differed by 1 SNP. The minimum pairwise SNP difference between a cattle and human isolate was >250 SNPs. The 10 study genomes were placed into a broader phylogenetic context by combining these with 151 lineage 1 *M*. *tuberculosis* genomes from Chennai (Fig. 2). This demonstrated that the 10 study isolates were distributed across the phylogeny, but that the 3 closely related isolate pairs remained clustered.

### Comparative genome analysis of cattle and human isolates

IS*6110* was mapped for each of the 10 study isolates and then compared between isolates from handlers and cattle (Supplementary Table 1). The IS*6110* copy number ranged from 1 to 4 (median 3) for isolates from humans, and 3 to 8 (median 6) for isolates from cattle. The 15 IS*6110* identified in the 6 isolates from humans were all intergenic and located in a conserved region of the genome upstream of the gene encoding CRISPR-associated endoribonuclease *cas2*. By comparison, the 23 IS*6110* identified in isolates from cattle were more widely distributed across the genome and 7 (30%) were intragenic (mostly in genes encoding hypothetical proteins). The number of SNPs in intragenic regions were 6 times higher than intergenic regions, a ratio that was comparable between human and animal-derived isolates (Fig. S1). Three of the four isolates from cattle had an IS*6110* insertion upstream of *espA* (Supplementary Table 2), which was not observed in isolates from handlers. *de novo* assembly of the 10 genomes and comparison of genetic content using core and pan-genome analyses demonstrated a core genome of ~3900 genes that comprised ~95% of genes present in any single isolate. No gene was identified that was uniquely present/absent in animal or human-derived isolates, and isolates were comparable in terms of the presence/absence of mycobacterial virulence factors listed in the Virulence Factor Database (Supplementary Table 3).

## Discussion

Several independent studies from the late 19th century onwards reported that *M*. *tuberculosis* was avirulent in cattle^13–15^, but recent reports of *M*. *tuberculosis* infection in livestock^2,5,6^ have led this to be experimentally re-evaluated. In a study published in 2010, cattle were infected with *M*. *tuberculosis* H37Rv or *M*. *bovis* 2122/97. Both species triggered strong cell-mediated immune responses, but post-mortem examination at 17 weeks only revealed visible pathology indicative of tuberculosis in *M*. *bovis*-infected animals. Despite the lack of pathology in the *M*. *tuberculosis*-infected group, 3/5 animals were culture positive, albeit at lower bacillary loads than *M*. *bovis*^14^. *M*. *tuberculosis* H37Rv was isolated in the early 1930s and has a long history of laboratory passage (and so could have become attenuated), which led to a second study that compared outcomes in cattle infected with *M*. *bovis* AF2122/97, *M*. *tuberculosis* H37Rv or *M*. *tuberculosis* BTB1558, which was isolated from a lymph node of a bull in Ethiopia in 2008^16^. Both isolates of *M*. *tuberculosis* caused comparable pathology, which was significantly lower than that caused by *M*. *bovis*^16^.

Despite these controlled experimental data, our findings of active *M*. *tuberculosis* infection in cattle associated with gross pathology shows that *M*. *tuberculosis* can both infect and cause pathology in a natural environment. Numerous lines of evidence confirmed that infection was due to *M*. *tuberculosis* rather than *M*. *bovis*, including whole genome sequence data that unequivocally placed cattle-associated isolates within a lineage that is the most common cause of human tuberculosis in southern India. Furthermore, the pathological findings on post-mortem examination performed shortly after death were consistent with active tuberculosis. This included visible pathology in the lung, spleen, lymph nodes and udder. There are several possible explanations for the discrepant findings between model systems and the natural environment. First, only some *M*. *tuberculosis* strains may be able to cause disease in cattle, including those not tested to date in experimental models. *M*. *tuberculosis* H37Rv and BTB1558 both reside in lineage 4 and are genetically distinct from the lineage 1 isolates identified in our study^17^. Second, in experimental models a single dose is given but in natural infection, repeated exposure is likely from infected handlers; route of infection may also be different. Third, experimental models are time limited but in natural infection, livestock may survive for more extended periods of time during which disease may develop and progress. There are also likely to be differences in the age of cattle; experimental animals are used at six months of age^16^, while livestock may reach a more advanced age prior to exposure. Finally, there could be variation in susceptibility between different cattle species.

The use of whole genome sequencing allowed us to examine the relatedness of *M*. *tuberculosis* from cattle and their handlers. We detected three isolate pairs that were highly related (≤4 SNPs different), of which two pairs were from handlers and one was from cattle. Based on this degree of relatedness, it is possible that *M*. *tuberculosis* transmission occurred between handlers, either directly or through an intermediate host. It is also possible that *M*. *tuberculosis* was transmitted between two cattle who shared a highly related strain, but an alternative possibility is that both animals were infected by a handler who was not captured in this study. We noted that the closest genetic distance between an isolate from cattle and humans was more than 250 SNPs different, which is consistent with an under-sampled population in which not all cases were captured at the time of our investigation. Further evidence for under-sampling was the isolation from cattle of a *M*. *tuberculosis* isolate that was multidrug-resistant, which was not detected in human isolates. The wider phylogenetic analysis including lineage 1 *M*. *tuberculosis* genomes from humans including those from Chennai revealed that isolates from our study were distributed across the phylogeny. The three highly related isolate pairs from our study each remained clustered but were in different parts of the tree, consistent with independent acquisition/transmission events.

The duration of infection in the 4 cattle is not known, but the gross pathology consistent with tuberculosis at the time of post-mortem examination suggests that they were infected for several years. IS6110 transposition is reportedly increased during TB infection in mice and after one year of laboratory culture^18^, which has led to the suggestion that IS*6110* transposition is dynamically adapted to the host and to adverse growth conditions. An investigation of IS*6110* in our study isolates demonstrated a modestly higher number of IS*6110* in the 4 cattle-derived versus 6 human-derived isolates and their location differed overall in human versus cattle-derived isolates. However, we note that our sample size was underpowered, and that previous reports have observed a higher copy number and intergenic locations of IS*6110* in clinical isolates^19,20^.

The Esx-1 secretion system is a major virulence factor that is essential for establishing a successful mycobacterial infection^21^. Expression of *esx*-1 is dependent on the transcription of the *espACD* operon, which in turn is regulated by EspR that binds the *espA* activating region (EAR) upstream of *espA*. Disruption in the EAR region has been linked to reduced expression of the operon. The EAR region is part of RD8 which is absent in *M*. *bovis* and leads to reduced expression of *esx-1* in *M*. *bovis* compared to *M*. *tuberculosis* lineages 1-4^22^. Variable regulation of *esx-1* in different lineages has been suggested to have evolved for the infection of different hosts^22^. The majority of the isolates from cattle in our study had an IS*6110* insertion in the EAR region while in isolates from cattle-handlers the region was intact, suggesting an adaptive change in response to infection in the cattle.

In conclusion, our findings confirm that advanced *M*. *tuberculosis* infection occurs in cattle. Further studies of *M*. *tuberculosis* in livestock are warranted in India to establish prevalence, further evaluate source attribution, and relate this to risks to public health.

## Methods

### Ethical approval, study design and sample collection

Ethical approval for the human and animal components of the study were obtained from the institutional ethics committees of National Institute for Research in Tuberculosis (NIRT, Chennai) and Tamil Nadu Veterinary and Animal Sciences University (TANUVAS, Chennai), respectively. Informed consent was obtained from livestock handlers and permission was obtained from farm managers to screen livestock and all the samples were processed according to the relevant guidelines and regulations.

A cross-sectional study was conducted on three cattle farms in Chennai, India between 2015 and 2016, where farm workers and their cattle were screened for tuberculosis. Only farm workers who were able to provide informed consent were included in the study. Farm workers were interviewed for symptoms of tuberculosis (cough for >2 weeks, fever, night sweats and/or weight loss). Two sputum samples were collected for bacteriological examination from farm workers with one or more symptoms. Cattle were simultaneously screened for tuberculosis using the CIT using standard methodology^23^. Sterile cotton swabs were used to collect nasal mucus samples from all CIT positive cattle, and ad hoc milk samples were collected into sterile containers from CIT positive cattle that were lactating (n = 17). Nasal and milk samples were processed for mycobacterial detection and culture, as described previously^24,25^. Opportunistic post-mortem examination was performed on CITpositive cattle that died during the study period. The respiratory, gastrointestinal and reproductive tracts and lymph nodes were examined for lesions of tuberculosis. Tissue samples were collected from lungs and placed into a sterile container for mycobacterial culture.

### Bacteriology and pathology

Human sputum samples were decontaminated and inoculated onto 2 LJ slopes and into two mycobacteria growth indicator (MGIT) tubes using standard methodology. The pellet was also inoculated onto a Lowenstein-Jensen (LJ) slope supplemented with sodium pyruvate. Slopes were incubated at 37 °C for up to 8 weeks, with weekly visual monitoring for mycobacterial growth. Positive growth was examined using ZN microscopy and the rapid strip based immunochromatographic test (ICT) (SD Bioline, Standard Diagnostics, Inc., Korea) for *M*. *tuberculosis* Complex, followed by spoligotyping.

Tissue samples taken from animals during post-mortem examination (~5 gm per sample) were rinsed with 5 ml sterile PBS and homogenised with a sterile Teflon grinding rod followed by centrifugation at 3500 rpm for 15 min. The pellet was resuspended with PBS with 5% sulphuric acid and centrifuged at 3500 rpm for 15 min. The pellet was resuspended in PBS and filtered through a sterile muslin cloth, ZN stained, and inoculated onto LJ slants (with or without glycerol and sodium pyruvate) and MGIT tubes supplemented with 800 μl of PANTA antibiotic mixtures. Positive growth in MGIT tubes was investigated by ZN staining to confirm the presence of mycobacteria, inoculated onto brain heart infusion (BHI) agar as a contamination check, and tested using the ICT to confirm *M*. *tuberculosis* Complex, followed by spoligotyping.

Drug susceptibility testing (DST) against four anti-TB drugs (streptomycin, isoniazid, rifampicin and ethambutol) was performed for all *M*. *tuberculosis*-positive cultures using the BACTEC MGIT 960 system.

### Genomic DNA extraction

Genomic DNA was extracted from *M*. *tuberculosis* isolates using the cetyl-trimethylammonium bromide (CTAB)/NaCl method^26^. Direct locus-DRa (0.2 µmol/µl) and DRb (0.2 µmol/µl) primers were used for spoligotyping^27^. The spacers between the direct repeats in the target region were amplified using two 18-nucleotide primers (DRb CCAAGAGGGGACGGAAAC and 5′ biotinylated DRa GGTTTTGGGTCTGACGAC). PCR products were hybridized onto a Biodyne C membrane (Isogen Bioscience, Maarsen, The Netherlands) containing immobilized synthetic oligomeric spacer sequences derived from the direct-repeat region of *M*. *tuberculosis* H37Rv and *M*. *bovis* BCG. Hybridized DNA was detected using an enhanced chemiluminescence kit (Biobasic, Israel), with exposure to X-ray film. The hybridization pattern was analysed using the SPOTCLUST database (http://tbinsight.cs.rpi.edu/run_spotclust.html).

### Whole genome sequencing and bioinformatics analysis

Whole genome paired-end sequencing was performed using Illumina HiSeq. 2500 instrument with a read length of 150 bp. Genome data have been deposited in the NCBI database under the Bioproject ID provided in Supplementary Table 2. Raw reads were filtered using Trimmomatic v0.36^28^ with parameters of minimum quality and length set to 20 and 60, respectively. The reference genome of H37Rv (NC_000962.3) was used for alignment using BWA v0.7.12^29^. A combination of Picard v2.2.4 (http://broadinstitute.github.io/picard/), GATK v3.5 and Samtools v1.3.1 were used to identify variants. SNPs with a mapping quality > 30, base quality > 50, and a depth of 5 were filtered. Additionally, each SNP was marked as homozygous if 80% or more reads supported the alternate allele, otherwise it was considered heterozygous. SNPs were annotated using custom python script (https://github.com/kumarnaren/mtb_vcf_annotator). Lineage determination was carried out based on lineage specific SNPs^12^ and RD analyzer v1.0^30^. A database of resistance conferring mutations was constructed by combining mutations reported previously^31–34^ and was used to detect resistance among the isolates. Filtered reads were also assembled using SPAdes v3.11.0^35^ with default parameters. Contigs greater than 500 bp in length were filtered for further analysis, annotated using Prokka v1.12^36^, and the resulting gff files were used for Roary v3.11.2^37^ for pangenome analysis. Virulence factors listed in the virulence factor database (VFDB) for *M*. *tuberculosis* H37Rv^38^ were downloaded and compared with the sequenced genomes using blastp with parameters of e-value > 0.00001, identity > 60% and coverage > 80%. The insertion sequence element IS6110 was analysed in genome sequences using the ISMapper tool with default settings^39^.

### Phylogenetic analysis

A pseudo-reference genome sequence for each isolate was generated by incorporating the isolate specific SNPs identified above. Repetitive regions reported by Holt *et al*.^40^ were masked using bedtools (v2.27.1). These pseudo-genomes were concatenated into a multi-fasta alignment file, which was used to identify variant sites using SNP-sites^41^. The multi-fasta output file was used as input to the random accelerated maximum likelihood (RAxML) tool^42^ to generate a phylogenetic tree based on the GTR-GAMMA algorithm with a bootstrap replication of 1000. The output tree was visualized and annotated using the online tool iTOL^43^. The output of SNP-sites was also used to calculate pairwise SNP difference. The sequenced genomes were placed into a wider genetic context by comparing the study genomes with 151 lineage 1 *M*. *tuberculosis* genomes generated during a previous study conducted in Chennai^44^. The 161 combined genomes were used to construct a phylogenetic tree using the method detailed above.

## Supplementary information

Dataset 1

## Data Availability

The sequence reads of the 10 study isolates have been submitted to NCBI under the Bioproject ID PRJNA512047 the assembled sequences were submitted to DDBJ/ENA/GenBank under the accessions SDUK00000000 - SDUT00000000.
