# Supplementary material for: Isolation and comparative genomics of Mycobacterium tuberculosis isolates from cattle and their attendants in South India
Source: Sci Rep. 2019 Nov 29;9:17892. doi: 10.1038/s41598-019-54268-x (PMC6884536; doi:10.1038/s41598-019-54268-x)
Supplement: Supplementary file 1 — Dataset 1 [file 41598_2019_54268_MOESM1_ESM.pdf]

Isolation and comparative genomics of *Mycobacterium tuberculosis* isolates from cattle and their attendants in SouthIndia

Kannan Palaniyandi[1,*,+], Narender Kumar[2,+], Maroudam Veerasamy[3], Ahmed Kabir Refaya[1], Chandrakumar Dolla[1], Subramanyam Balaji[1], Dhanaraj Baskaran[1], Kannan Thiruvengadam[1], Ananthi Rajendran[1], Sujatha Narayanan[1], Dhinakar Raj[3], Soumya Swaminathan[4], and Sharon J. Peacock[2,5]

Supplementary Figure 1: Genetic variations identified among the genomes and plotted as groups: Animal; isolates obtianed from cattle and Human; isolates obtained from farm-workers.

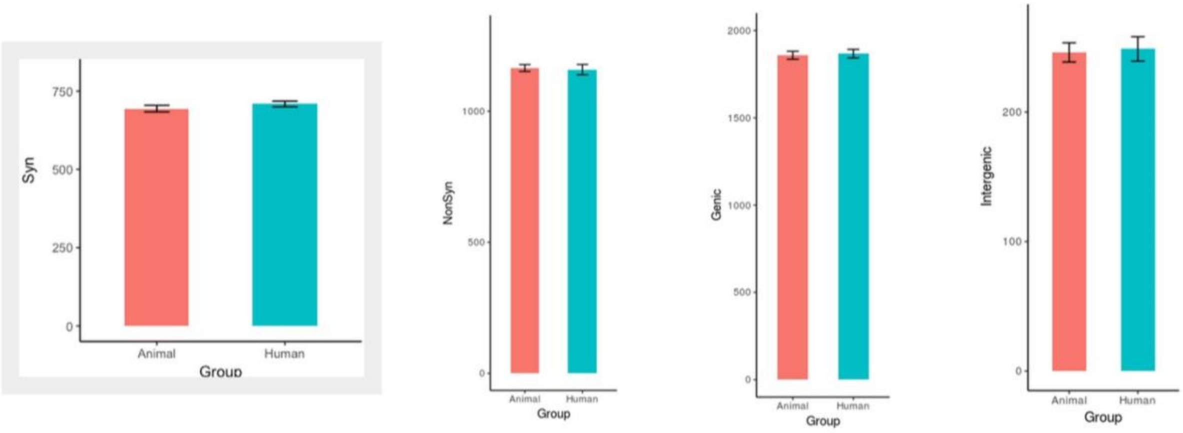

A) Synonymous mutaions

B) Non-Synonymous mutations

C) Intragenic mutations

D) Intergenic mutaions

Supplementary Table 1. IS6110 insertion sites identified among the genomes.

The ISMapper tool was used to identify the insertion sites of the IS*6110* insertion element. "X" and "Y" represent the left and right side of the insertion site identified in each genome.

Strain id	Source	X	Y	Left gene (Rv ID)	Right gene (Rv ID)	Genic/Intergenic
**AH03**	Human	3120177	3120212	hypothetical protein (Rv2813)	CRISPR-associated endoribonuclease *cas2* (Rv2816c)	intergenic
		3120540	3121857	hypothetical protein (Rv2813)	CRISPR-associated endoribonuclease *cas2* (Rv2816c)	intergenic
		3119408	3119223	hypothetical protein (Rv2813)	CRISPR-associated endoribonuclease *cas2* (Rv2816c)	intergenic
**AH29**	Human	3119223	3119408	hypothetical protein (Rv2813)	CRISPR-associated endoribonuclease *cas2* (Rv2816c)	intergenic
		3120169	3120212	hypothetical protein (Rv2813)	CRISPR-associated endoribonuclease *cas2* (Rv2816c)	intergenic
		3122380	3122521	hypothetical protein (Rv2813)	CRISPR-associated endoribonuclease *cas2* (Rv2816c)	intergenic
		3123466	3123353	hypothetical protein (Rv2813)	CRISPR-associated endoribonuclease *cas2* (Rv2816c)	intergenic
**AH69**	Human	3120540	3121856	hypothetical protein (Rv2813)	CRISPR-associated endoribonuclease *cas2* (Rv2816c)	intergenic
**AH85**	Human	3119223	3119408	hypothetical protein (Rv2813)	CRISPR-associated endoribonuclease *cas2* (Rv2816c)	intergenic
		3120177	3120212	hypothetical protein (Rv2813)	CRISPR-associated endoribonuclease *cas2* (Rv2816c)	intergenic
		3123466	3123354	hypothetical protein (Rv2813)	CRISPR-associated endoribonuclease *cas2* (Rv2816c)	intergenic
**AH90**	Human	3119223	3119408	hypothetical protein (Rv2813)	CRISPR-associated endoribonuclease *cas2* (Rv2816c)	intergenic
		3120177	3120212	hypothetical protein (Rv2813)	CRISPR-associated endoribonuclease *cas2* (Rv2816c)	intergenic
		3123466	3123350	hypothetical protein (Rv2813)	CRISPR-associated endoribonuclease *cas2* (Rv2816c)	intergenic
**AH91**	Human	3120513	3121874	hypothetical protein (Rv2813)	CRISPR-associated endoribonuclease *cas2* (Rv2816c)	intergenic
**KH126**	Animal	1895487	1895382	Rv1667c	Rv1667c	genic
		2041598	2041665	*ppe28* (Rv1800)	*ppe29* (Rv1801)	intergenic

		2368569	2368526	*ppe36* (Rv2108)	*prcA* (Rv2109c)	intergenic
		3120521	3121898	hypothetical protein (Rv2813)	CRISPR-associated endoribonuclease *cas2* (Rv2816c)	intergenic
		4056795	4056802	*espA* (Rv3616c)	*ephA* (Rv3617)	intergenic
**KH127**	Animal	888788	888796	oxidoreductase (Rv0794c)	insertion sequence element IS1547 transposase (Rv0797)	intergenic
		889020	890443	oxidoreductase (Rv0794c)	insertion sequence element IS1547 transposase (Rv0797)	intergenic
		1895413	1895394	Rv1667c	Rv1667c	genic
		1995677	1995767	hypothetical protein (Rv1761c)	hypothetical protein (Rv1761c)	genic
		1999316	1999263	hypothetical protein (Rv1761c)	hypothetical protein (Rv1766)	intergenic
		3120499	3121922	hypothetical protein (Rv2813)	CRISPR-associated endoribonuclease *cas2* (Rv2816c)	intergenic
		3711993	3711772	transposase fusion protein (Rv3327)	transposase fusion protein (Rv3327)	genic
		4056783	4056802	*espA* (Rv3616c)	*ephA* (Rv3617)	intergenic
**KH128**	Animal	888783	888786	oxidoreductase (Rv0794c)	insertion sequence element IS1547 transposase (Rv0797)	intergenic
		1995761	1995764	hypothetical protein (Rv1761c)	hypothetical protein (Rv1761c)	genic
		1999271	1999275	hypothetical protein (Rv1761c)	hypothetical protein (Rv1766)	intergenic
		3119408	3119223	hypothetical protein (Rv2813)	CRISPR-associated endoribonuclease *cas2* (Rv2816c)	intergenic
		3119551	3119449	hypothetical protein (Rv2813)	CRISPR-associated endoribonuclease *cas2* (Rv2816c)	intergenic
		3120212	3120177	hypothetical protein (Rv2813)	CRISPR-associated endoribonuclease *cas2* (Rv2816c)	intergenic
		4056799	4056803	*espA* (Rv3616c)	*ephA* (Rv3617)	Intergenic
**KH143**	Animal	3120538	3121866	hypothetical protein (Rv2813)	CRISPR-associated endoribonuclease *cas2* (Rv2816c)	intergenic
		4197857	4197790	hypothetical protein (Rv3749c)	hypothetical protein (Rv3749c)	genic
		1889840	1889683	*pks9* (Rv1664)	*pks9* (Rv1664)	genic

Supplementary Table 2. Sequence data metrics for the 10 *M. tuberculosis* isolates used in the study.

Strain Id	Source	Strain name (NCBI)	NCBI Bioproject id	Reads ($x10^6$)	Contigs (>500b)	rRNA	tRNA	CDS	N50	Rd analysis	Lineage
**AH03**	Human	NIRTAH01	PRJNA512047	5.2	81	3	45	4120	129915	Rd239	L1
**AH29**	Human	NIRTAH02	PRJNA512047	10.2	91	3	45	4138	110988	Rd239	L1
**AH69**	Human	NIRTAH03	PRJNA512047	5.6	82	3	45	4118	129914	Rd239	L1
**AH85**	Human	NIRTAH04	PRJNA512047	16.7	89	3	45	4136	129915	Rd239	L1
**AH90**	Human	NIRTAH05	PRJNA512047	16.1	88	3	45	4127	125613	Rd239	L1
**AH91**	Human	NIRTAH06	PRJNA512047	1.3	96	3	45	4140	125590	Rd239	L1
**KH126**	Animal	NIRTAH07	PRJNA512047	1.2	101	3	45	4158	106348	Rd239	L1
**KH127**	Animal	NIRTAH08	PRJNA512047	1.1	87	3	45	4138	129915	Rd239	L1
**KH128**	Animal	NIRTAH09	PRJNA512047	5.0	87	3	45	4127	129915	Rd239	L1
**KH143**	Animal	NIRTAH10	PRJNA512047	5.6	119	3	45	4168	94243	Rd239	L1

Supplementary Table 3. Presence of virulence factors in the 10 study *M. tuberculosis* isolates. Virulence factors listed in VFDB database for H37Rv and presence of absence in the 10 study isolates. First column represents that VFDB ID as given in the database. 1, present; 0, absent.

VFDB ID	KH126	KH127	KH128	KH143	AH29	AH03	AH69	AH85	AH90	AH91
**VFG009967**	1	1	1	1	1	1	1	1	1	1
**VFG002384**	1	1	1	1	1	1	1	1	1	1
**VFG001405**	1	1	1	1	1	1	1	1	1	1
**VFG031669**	1	1	1	1	1	1	1	1	1	1
**VFG030480**	1	1	1	1	1	1	1	1	1	1
**VFG007980**	1	1	1	1	1	1	1	1	1	1
**VFG010034**	1	1	1	1	1	1	1	1	1	1
**VFG030452**	1	1	1	1	1	1	1	1	1	1
**VFG029839**	1	1	1	1	1	1	1	1	1	1
**VFG009934**	1	1	1	1	1	1	1	1	1	1
**VFG007878**	1	1	1	1	1	1	1	1	1	1
**VFG031722**	1	1	1	1	1	1	1	1	1	1
**VFG002393**	1	1	1	1	1	1	1	1	1	1
**VFG007866**	1	1	1	1	1	1	1	1	1	1
**VFG001812**	1	1	1	1	1	1	1	1	1	1
**VFG001818**	1	1	1	1	1	1	1	1	1	1
**VFG008258**	1	1	1	1	1	1	1	1	1	1
**VFG001825**	1	1	1	1	1	1	1	1	1	1
**VFG007953**	1	1	1	1	1	1	1	1	1	1
**VFG001419**	1	1	1	1	1	1	1	1	1	1
**VFG009249**	1	1	1	1	1	1	1	1	1	1
**VFG002395**	1	1	1	1	1	1	1	1	1	1
**VFG001824**	1	1	1	1	1	1	1	1	1	1

**VFG001406**	1	1	1	1	1	1	1	1	1	1
**VFG022130**	1	1	1	1	1	1	1	1	1	1
**VFG002399**	1	1	1	1	1	1	1	1	1	1
**VFG001815**	1	1	1	1	1	1	1	1	1	1
**VFG007974**	1	1	1	1	1	1	1	1	1	1
**VFG031808**	1	1	1	1	1	1	1	1	1	1
**VFG017480**	1	1	1	1	1	1	1	1	1	1
**VFG030148**	1	1	1	1	1	1	1	1	1	1
**VFG022224**	1	1	1	1	1	1	1	1	1	1
**VFG009645**	1	1	1	1	1	1	1	1	1	1
**VFG031591**	1	1	1	1	1	1	1	1	1	1
**VFG002387**	1	1	1	1	1	1	1	1	1	1
**VFG001418**	1	1	1	1	1	1	1	1	1	1
**VFG007823**	0	1	0	1	1	1	1	1	1	1
**VFG031840**	1	1	1	1	1	1	1	1	1	1
**VFG009947**	1	1	1	1	1	1	1	1	1	1
**VFG029383**	1	1	1	1	1	1	1	1	1	1
**VFG010022**	1	1	1	1	1	1	1	1	1	1
**VFG001409**	1	1	1	1	1	1	1	1	1	1
**VFG002388**	1	1	1	1	1	1	1	1	1	1
**VFG001813**	1	1	1	1	1	1	1	1	1	1
**VFG009977**	1	1	1	1	1	1	1	1	1	1
**VFG009767**	0	0	0	0	0	0	0	0	0	0
**VFG043448**	1	1	1	1	1	1	1	1	1	1
**VFG030286**	1	1	1	1	1	1	1	1	1	1
**VFG001390**	1	1	1	1	1	1	1	1	1	1
**VFG002397**	1	1	1	1	1	1	1	1	1	1
**VFG022303**	0	0	0	0	0	0	0	0	0	0
**VFG001811**	1	1	1	1	1	1	1	1	1	1
**VFG030983**	1	1	1	1	1	1	1	1	1	1

**VFG010070**	1	1	1	1	1	1	1	1	1	1
**VFG044071**	1	1	1	1	1	1	1	1	1	1
**VFG007918**	1	1	1	1	1	1	1	1	1	1
**VFG022019**	1	1	1	1	1	1	1	1	1	1
**VFG031564**	0	0	0	0	0	0	0	0	0	0
**VFG030562**	1	1	1	1	1	1	1	1	1	1
**VFG030880**	1	1	1	1	1	1	1	1	1	1
**VFG009579**	1	1	1	1	1	1	1	1	1	1
**VFG001814**	1	1	1	1	1	1	1	1	1	1
**VFG001391**	1	1	1	1	1	1	1	1	1	1
**VFG028201**	1	1	1	1	1	1	1	1	1	1
**VFG022114**	1	1	1	1	1	1	1	1	1	1
**VFG010161**	1	1	1	1	1	1	1	1	1	1
**VFG001820**	1	1	1	1	1	1	1	1	1	1
**VFG001810**	1	1	1	1	1	1	1	1	1	1
**VFG001383**	1	1	1	1	1	1	1	1	1	1
**VFG029628**	0	0	0	1	0	0	0	0	0	0
**VFG001408**	1	1	1	1	1	1	1	1	1	1
**VFG022066**	1	1	1	1	1	1	1	1	1	1
**VFG043447**	1	1	1	1	1	0	1	1	1	1
**VFG007965**	1	1	1	1	1	1	1	1	1	1
**VFG010235**	1	1	1	1	1	1	1	1	1	1
**VFG030107**	1	1	1	1	1	1	1	1	1	1
**VFG001407**	1	1	1	1	1	1	1	1	1	1
**VFG001381**	1	1	1	1	1	1	1	1	1	1
**VFG001388**	1	1	1	1	1	1	1	1	1	1
**VFG002401**	1	1	1	1	1	1	1	1	1	1
**VFG030509**	1	1	1	1	1	1	1	1	1	1
**VFG002378**	1	1	1	1	1	1	1	1	1	1
**VFG030230**	1	1	1	1	1	1	1	1	1	1

**VFG009561**	1	1	1	1	1	1	1	1	1	1
**VFG010249**	1	1	1	1	1	1	1	1	1	1
**VFG010058**	1	1	1	1	1	1	1	1	1	1
**VFG010221**	1	1	1	1	1	1	1	1	1	1
**VFG031777**	1	1	1	1	1	1	1	1	1	1
**VFG001403**	1	1	1	1	1	1	1	1	1	1
**VFG002386**	1	1	1	1	1	1	1	1	1	1
**VFG007849**	1	1	1	1	1	1	1	1	1	1
**VFG001413**	1	1	1	1	1	1	1	1	1	1
**VFG009987**	1	1	1	1	1	1	1	1	1	1
**VFG001817**	1	1	1	1	1	1	1	1	1	1
**VFG002402**	1	1	1	1	1	1	1	1	1	1
**VFG029987**	1	1	1	1	1	1	1	1	1	1
**VFG001823**	1	1	1	1	1	1	1	1	1	1
**VFG001826**	1	1	1	1	1	1	1	1	1	1
**VFG001393**	1	1	1	1	1	1	1	1	1	1
**VFG007832**	1	1	1	1	1	1	1	1	1	1
**VFG001394**	1	1	1	1	1	1	1	1	1	1
**VFG001821**	1	1	1	1	1	1	1	1	1	1
**VFG002398**	1	1	1	1	1	1	1	1	1	1
**VFG007999**	1	1	1	1	1	1	1	1	1	1
**VFG022162**	1	1	1	1	1	1	1	1	1	1
**VFG022082**	1	1	1	1	1	1	1	1	1	1
**VFG031877**	1	1	1	1	1	1	1	1	1	1
**VFG021940**	1	1	1	1	1	1	1	1	1	1
**VFG010010**	1	1	1	1	1	1	1	1	1	1
**VFG031643**	1	1	1	1	1	1	1	1	1	1
**VFG001395**	1	1	1	1	1	1	1	1	1	1
**VFG008228**	1	1	1	1	1	1	1	1	1	1
**VFG030725**	1	1	1	1	1	1	1	1	1	1

**VFG031390**	1	1	1	1	1	1	1	1	1	1
**VFG001421**	1	1	1	1	1	1	1	1	1	1
**VFG030325**	1	1	1	1	1	1	1	1	1	1
**VFG001414**	1	1	1	1	1	1	1	1	1	1
**VFG010093**	1	1	1	1	1	1	1	1	1	1
**VFG029761**	1	1	1	1	1	1	1	1	1	1
**VFG022333**	1	1	1	1	1	1	1	1	1	1
**VFG043446**	1	1	1	1	1	1	1	1	1	1
**VFG030932**	0	0	0	0	0	0	0	0	0	0
**VFG002380**	1	1	1	1	1	1	1	1	1	1
**VFG030754**	1	1	1	1	1	1	1	1	1	1
**VFG007904**	1	1	1	1	1	1	1	1	1	1
**VFG001397**	1	1	1	1	1	1	1	1	1	1
**VFG007744**	1	1	1	1	1	1	1	1	1	1
**VFG031296**	1	1	1	1	1	1	1	1	1	1
**VFG009957**	1	1	1	1	1	1	1	1	1	1
**VFG044070**	1	1	1	1	1	1	1	1	1	1
**VFG030671**	1	1	1	1	1	1	1	1	1	1
**VFG001410**	1	1	1	1	1	1	1	1	1	1
**VFG031348**	1	1	1	1	1	1	1	1	1	1
**VFG001379**	1	1	1	1	1	1	1	1	1	1
**VFG022265**	1	1	1	1	1	1	1	1	1	1
**VFG022146**	1	1	1	1	1	1	1	1	1	1
**VFG001415**	1	1	1	1	1	1	1	1	1	1
**VFG010046**	1	1	1	1	1	1	1	1	1	1
**VFG030258**	1	1	1	1	1	1	1	1	1	1
**VFG002390**	1	1	1	1	1	1	1	1	1	1
**VFG021972**	1	1	1	1	1	1	1	1	1	1
**VFG029483**	1	1	1	1	1	1	1	1	1	1
**VFG031085**	1	1	1	1	1	1	1	1	1	1

**VFG029936**	1	1	1	1	1	1	1	1	1	1
**VFG022051**	1	1	1	1	1	1	1	1	1	1
**VFG008136**	1	1	1	1	1	1	1	1	1	1
**VFG010277**	1	1	1	1	1	1	1	1	1	1
**VFG029888**	1	1	1	1	1	1	1	1	1	1
**VFG022035**	1	1	1	1	1	1	1	1	1	1
**VFG044072**	1	1	1	1	1	1	1	1	1	1
**VFG022098**	1	1	1	1	1	1	1	1	1	1
**VFG008007**	1	1	1	1	1	1	1	1	1	1
**VFG031242**	1	1	1	1	1	1	1	1	1	1
**VFG001389**	1	1	1	1	1	1	1	1	1	1
**VFG001398**	1	1	1	1	1	1	1	1	1	1
**VFG028200**	1	1	1	1	1	1	1	1	1	1
**VFG001382**	1	1	1	1	1	1	1	1	1	1
**VFG030356**	1	1	1	1	1	1	1	1	1	1
**VFG001385**	1	1	1	1	1	1	1	1	1	1
**VFG030817**	1	1	1	1	1	1	1	1	1	1
**VFG030052**	1	1	1	1	1	1	1	1	1	1
**VFG009997**	1	1	1	1	1	1	1	1	1	1
**VFG001399**	1	1	1	1	1	1	1	1	1	1
**VFG007959**	1	1	1	1	1	1	1	1	1	1
**VFG002391**	1	1	1	1	1	1	1	1	1	1
**VFG024776**	1	1	1	1	1	1	1	1	1	1
**VFG021827**	1	1	1	1	1	1	1	1	1	1
**VFG002385**	1	1	1	1	1	1	1	1	1	1
**VFG030616**	1	1	1	1	1	1	1	1	1	1
**VFG001412**	1	1	1	1	1	1	1	1	1	1
**VFG002392**	1	1	1	1	1	1	1	1	1	1
**VFG010138**	1	1	1	1	1	1	1	1	1	1
**VFG021988**	1	1	1	1	1	1	1	1	1	1

**VFG024724**	1	1	1	1	1	1	1	1	1	1
**VFG031457**	1	1	1	1	1	1	1	1	1	1
**VFG030423**	1	1	1	1	1	1	1	1	1	1
**VFG029681**	1	1	1	1	1	1	1	1	1	1
**VFG009570**	1	1	1	1	1	1	1	1	1	1
**VFG001392**	1	1	1	1	1	1	1	1	1	1
**VFG010263**	1	1	1	1	1	1	1	1	1	1
**VFG009382**	1	1	1	1	1	1	1	1	1	1
**VFG001411**	1	1	1	1	1	1	1	1	1	1
**VFG001404**	1	1	1	1	1	1	1	1	1	1
**VFG031590**	1	1	1	1	1	1	1	1	1	1
**VFG001819**	1	1	1	1	1	1	1	1	1	1
**VFG007946**	1	1	1	1	1	1	1	1	1	1
**VFG010115**	1	1	1	1	1	1	1	1	1	1
**VFG001386**	1	1	1	1	1	1	1	1	1	1
**VFG009810**	1	1	1	1	1	1	1	1	1	1
**VFG009869**	1	1	1	1	1	1	1	1	1	1
**VFG002389**	1	1	1	1	1	1	1	1	1	1
**VFG009553**	1	1	1	1	1	1	1	1	1	1
**VFG002383**	1	1	1	1	1	1	1	1	1	1
**VFG009640**	1	1	1	1	1	1	1	1	1	1
**VFG022004**	1	1	1	1	1	1	1	1	1	1
**VFG001816**	1	1	1	1	1	1	1	1	1	1
**VFG002379**	1	1	1	1	1	1	1	1	1	1
**VFG001384**	1	1	1	1	1	1	1	1	1	1
**VFG031191**	1	1	1	1	1	1	1	1	1	1
**VFG001380**	1	1	1	1	1	1	1	1	1	1
**VFG030203**	1	1	1	1	1	1	1	1	1	1
**VFG030388**	1	1	1	1	1	1	1	1	1	1
**VFG001822**	1	1	1	1	1	1	1	1	1	1

**VFG021840**	1	1	1	1	1	1	1	1	1	1
**VFG002396**	1	1	1	1	1	1	1	1	1	1
**VFG002381**	1	1	1	1	1	1	1	1	1	1
**VFG029570**	0	0	0	0	0	0	0	0	0	0
**VFG030790**	1	1	1	1	1	1	1	1	1	1
**VFG022340**	1	1	1	1	1	1	1	1	1	1
**VFG007991**	1	1	1	1	1	1	1	1	1	1
**VFG010184**	1	1	1	1	1	1	1	1	1	1
**VFG002403**	1	1	1	1	1	1	1	1	1	1
**VFG007857**	1	1	1	1	1	1	1	1	1	1
**VFG031138**	1	1	1	1	1	1	1	1	1	1
**VFG043550**	1	1	1	1	1	1	1	1	1	1
**VFG010207**	1	1	1	1	1	1	1	1	1	1
**VFG002400**	1	1	1	1	1	1	1	1	1	1
**VFG028202**	1	1	1	1	1	1	1	1	1	1
**VFG030816**	1	1	1	1	1	1	1	1	1	1
**VFG002382**	1	1	1	1	1	1	1	1	1	1
**VFG009839**	1	1	1	1	1	1	1	1	1	1
**VFG031511**	1	1	1	1	1	1	1	1	1	1
**VFG021800**	0	0	0	0	0	0	0	0	0	0
**VFG031035**	1	1	1	1	1	1	1	1	1	1
**VFG001420**	1	1	1	1	1	1	1	1	1	1
**VFG028199**	1	1	1	1	1	1	1	1	1	1
**VFG001396**	1	1	1	1	1	1	1	1	1	1
